# Allergic profile of Congolese individuals exposed to flour dust as compared with a non-exposed work group

**DOI:** 10.1186/2045-7022-3-S2-P9

**Published:** 2013-07-16

**Authors:** Dieudonné Nyembue Tshipukane, Eliseé Kembia, Léonie Lusamba, Marie Jeanne Nkoy, Boniface Kamanga, Jean-Marie Kayembe, Jeroen Vanoirbeek, Hans Scheers, Frank Buntinx, Peter Hellings, Mark Jorissen

**Affiliations:** 1 University of Kinshasa, Otorhinolaryngology Department, Kinshasa, Congo; 2 Hopital Sino-Congolais, ENT service, Kinshasa, Congo; 3 University of Kinshasa, Médecine Physique, Kinshasa, Congo; 4 University of Kinshasa, Pneumologie, Kinshasa, Congo; 5 KU Leuven, Occupational, Environmental Medicine, Leuven, Belgium; 6 KU Leuven, Academic Center for General Practice, Leuven, Belgium; 7 KU Leuven, Experimental Oto-rhino-laryngology, Leuven, Belgium

## Background

Airway symptoms are common among workers exposed to flour dust. However, no such evaluation had been done in Kinshasa.

## Objective

To study the prevalence of airway symptoms, sensitization profile and risk factors of allergic disease in Congolese workers exposed to flour dust.

## Methods

A cross-sectional study was performed among 263 workers directly exposed to flour (wheat, manioc and/or maize), 278 indirectly exposed to wheat flour and 268 controls. Indirect exposure was defined as administrative workers of the bakeries. Individual rhinitis and asthma symptoms were asked for, and skin prick tests (SPT), peak nasal inspiratory flow and pulmonary function performed.

## Results

Workers that were directly exposed to flour dust showed a prevalence of rhinitis (57.4%), rhinoconjonctivitis (43.5%), and nocturnal cough (11.0%) significantly higher than the indirectly exposed (43.5%, 14.7% and 5.4%) and controls (37.3%, 11.6% and 6.3%), all p<0.05. 36.8% of flour dust directly exposed workers showed positive SPT to at least one allergens, that was significantly lower than 44.2% of controls. DPT and cockroaches were the most prevalent allergens in all groups. Storage mite was more prevalent among workers directly exposed, while pollen mix, sunflower pollen and crab were more prevalent in controls. Within the directly exposed group, sensitization to manioc flour was higher among individuals exposed to manioc and/or maize flour than those exposed to wheat flour. Of individuals reporting rhinitis, 37.0% showed positive SPT results. In multivariate analysis, direct exposure to flour, neighborhood of flour mill and mice in the house significantly increased the risk of having airway symptoms. Mice in the house increased the risk of sensitization. However, cooking with electricity was negatively associated with both airway symptoms and sensitization.

## Conclusion

Although sensitization was lower in the directly exposed group, this study showed that flour dust constitutes an increased risk for having airway symptoms compared to controls. Flour dust control measures is needed at workplace.

